# Supplementary material for: A Likelihood Approach for Real-Time Calibration of Stochastic Compartmental Epidemic Models
Source: PLoS Comput Biol. 2017 Jan 17;13(1):e1005257. doi: 10.1371/journal.pcbi.1005257 (PMC5240920; doi:10.1371/journal.pcbi.1005257)
Supplement: S1 File — (TAR.GZ) [file pcbi.1005257.s014.tar.gz › HSPH_Online-SI-Revision/EnKF07/n100-severe/n100-severe_EnKF_table-8.pdf]

1	R0	{1.65278, 9.06793}	{2.32607, 8.89594}	{{0.408794, 2.70128}, {3., 13.7208}}	{69.3394, 48.9987}	inf	990.	4388.05	{2782.37, 2782.37}	343.238	0.496185	newly	963.	3053.46	{1142., 4006.}	217.078	0.341999	Reff	1.60825	0.375594	{0.0746715, 0.650724}	76.8565	ar	66 342.	176 587.	{170 612., 174 962.}	166.177
2	R0	{1.4321, 3.80644}	{2.17147, 6.13013}	{{0.849237, 2.17253}, {3., 10.0948}}	{76.717, 80.7919}	inf	86.	33 841.7	{13 334.1, 67 531.4}	39 251.2	2.49206	newly	223.	42 255.7	{10 247., 88 208.}	18 850.4	2.14392	Reff	1.42869	1.12626	{0.404311, 1.72321}	34.2356	ar	54 217.	143 283.	{92 262., 204 572.}	165.296
3	R0	{1.68388, 4.76028}	{2.08148, 10.8216}	{{0.768935, 3.6103}, {3., 17.0568}}	{46.8886, 139.125}	inf	40.	25 051.9	{5779.77, 46 815.6}	62 529.8	2.63542	newly	115.	23 140.	{5111., 57 341.}	20 021.7	2.07048	Reff	1.68211	1.3206	{0.288763, 2.22219}	34.6368	ar	68 299.	129 097.	{84 178., 173 665.}	91.7237
4	R0	{1.66014, 6.95383}	{2.53656, 7.31843}	{{2.16008, 4.31434}, {3., 14.7055}}	{54.1317, 43.4116}	inf	267.	23 733.3	{3530.89, 56 894.8}	8789.68	1.78485	newly	379.	27 796.5	{2115., 71 784.}	7234.89	1.634	Reff	1.65028	1.06099	{0.329848, 2.18569}	50.2337	ar	66 820.	161 840.	{97 621., 235 056.}	144.472
5	R0	{1.69984, 11.2038}	{2.25663, 9.05188}	{{0.401808, 2.6935}, {3., 13.6936}}	{67.847, 40.4163}	inf	1644.	4575.94	{2773.03, 2773.03}	179.057	0.287545	newly	1210.	3180.22	{1140., 4007.}	164.96	0.256233	Reff	1.6312	0.380888	{0.0734219, 0.651654}	76.8099	ar	68 462.	177 809.	{170 714., 181 863.}	159.719
6	R0	{1.65876, 6.66217}	{2.49878, 7.43629}	{{2.15944, 4.30788}, {3., 14.7082}}	{55.3684, 45.2223}	inf	148.	22 412.9	{7025.97, 41 229.2}	15 043.9	2.0573	newly	251.	24 354.	{5739., 63 383.}	9602.77	1.79985	Reff	1.65251	0.926587	{0.306124, 2.13456}	52.877	ar	67 105.	148 426.	{82 941., 226 865.}	123.501
7	R0	{1.69966, 5.89996}	{2.34938, 5.36499}	{{0.849062, 2.17257}, {3., 8.46695}}	{60.679, 39.7934}	inf	95.	31 621.9	{11 901.6, 61 185.1}	33 188.1	2.3628	newly	161.	42 088.8	{11 378., 89 770.}	26 045.1	2.23023	Reff	1.69633	1.14628	{0.356372, 1.82008}	39.919	ar	69 396.	136 686.	{76 340., 197 355.}	101.278
8	R0	{1.53428, 6.3734}	{2.03637, 4.20668}	{{0.086665, 0.937852}, {3., 5.82208}}	{78.0092, 44.8168}	inf	389.	5376.02	{549.807, 16 998.}	1283.63	0.857145	newly	553.	6503.69	{479., 16 867.}	1083.62	0.734816	Reff	1.51552	0.311858	{0.0127996, 0.452893}	79.4715	ar	59 855.	170 766.	{157 345., 220 231.}	187.057
9	R0	{1.58939, 3.48608}	{2.17265, 9.63805}	{{1.33087, 1.99301}, {3., 17.0587}}	{50.0873, 188.828}	inf	10.	25 891.4	{5883.38, 64 116.8}	258 814.	3.2689	newly	29.	27 960.4	{5260., 83 297.}	96 315.3	2.75095	Reff	1.58888	1.35375	{0.383276, 2.22217}	34.8857	ar	62 575.	130 312.	{77 348., 178 956.}	109.421
10	R0	{1.58528, 5.5593}	{2.42577, 6.83879}	{{1.46514, 3.39094}, {3., 10.2973}}	{56.9368, 50.1842}	inf	179.	2318.14	{1471.37, 2611.35}	1195.05	0.991039	newly	327.	3260.05	{784., 3353.}	898.309	0.691251	Reff	1.57528	0.568526	{0.217752, 0.990787}	64.8469	ar	63 039.	181 786.	{169 707., 208 886.}	188.371
11	R0	{1.58305, 4.62721}	{2.29842, 5.96896}	{{0.847586, 2.17241}, {3., 10.0924}}	{67.3523, 59.7669}	inf	50.	31 467.7	{11 890.7, 61 044.4}	62 835.4	2.64706	newly	133.	40 010.8	{12 339., 89 971.}	29 985.8	2.28422	Reff	1.57961	1.07381	{0.393498, 1.81843}	41.7506	ar	63 222.	139 164.	{84 529., 197 241.}	122.937
12	R0	{1.70234, 9.34626}	{2.20992, 8.77198}	{{0.414603, 2.70931}, {3., 13.7276}}	{64.5907, 49.1728}	inf	872.	4975.5	{2792.13, 2792.13}	470.585	0.562104	newly	836.	3357.41	{1145., 4014.}	301.604	0.421529	Reff	1.66487	0.356302	{0.0753944, 0.559901}	78.5988	ar	68 213.	177 347.	{170 940., 174 881.}	159.991
13	R0	{1.68556, 9.74954}	{2.35333, 8.99359}	{{0.40606, 2.70312}, {3., 13.7035}}	{68.4574, 45.7255}	inf	1232.	4600.36	{2783.03, 2783.03}	273.584	0.411618	newly	1100.	3198.96	{1165., 3965.}	190.867	0.292226	Reff	1.63051	0.382441	{0.0742109, 0.651254}	76.7132	ar	68 426.	177 139.	{170 635., 181 878.}	158.876
14	R0	{1.59876, 9.78897}	{2.17637, 8.96301}	{{0.408874, 2.69856}, {3., 13.7276}}	{71.8086, 46.1236}	inf	1006.	4357.57	{2779.69, 2779.69}	333.173	0.484113	newly	885.	2984.92	{1147., 3950.}	237.279	0.368603	Reff	1.55367	0.356352	{0.0739159, 0.650327}	77.0639	ar	63 553.	176 146.	{170 446., 175 082.}	177.163
15	R0	{1.43577, 5.12819}	{2.48364, 5.0547}	{{1.2207, 1.29206}, {3., 7.54537}}	{76.2194, 48.4731}	inf	217.	2779.16	{735.003, 3082.32}	1184.94	0.810058	newly	384.	3914.68	{661., 6410.}	925.599	0.619032	Reff	1.42469	0.524235	{0.178209, 1.20131}	69.1314	ar	53 259.	181 505.	{167 202., 202 326.}	240.798
16	R0	{1.58866, 8.73191}	{2.45374, 6.65735}	{{1.46386, 3.37773}, {3., 10.2938}}	{58.5299, 38.702}	inf	604.	4371.93	{1510.06, 8792.83}	626.74	0.563263	newly	587.	5467.68	{772., 25 500.}	833.329	0.549142	Reff	1.56477	0.622544	{0.0220689, 1.46298}	62.6193	ar	64 349.	188 210.	{170 293., 245 522.}	192.483
17	R0	{1.43012, 3.8163}	{2.27083, 5.31129}	{{0.849722, 2.17256}, {3., 8.19614}}	{79.233, 64.7072}	inf	64.	32 225.6	{8665.2, 61 072.7}	50 252.5	2.58339	newly	190.	43 471.2	{13 007., 88 272.}	22 780.7	2.22592	Reff	1.42695	1.15705	{0.490381, 1.72278}	30.9644	ar	53 353.	143 646.	{98 804., 197 391.}	170.241
18	R0	{1.47965, 5.16472}	{2.44603, 4.4457}	{{1.76903, 1.25864}, {3., 12.7812}}	{66.429, 67.0371}	inf	233.	25 874.6	{4856.05, 58 748.}	11 006.2	1.84564	newly	443.	31 893.7	{3977., 78 528.}	7101.36	1.61423	Reff	1.47015	0.595571	{0.240531, 1.36529}	60.911	ar	56 086.	162 478.	{78 937., 248 489.}	189.954
19	R0	{1.57191, 5.2314}	{2.46498, 5.01393}	{{1.764, 1.2393}, {3., 12.8227}}	{58.8011, 74.9976}	inf	225.	27 400.	{4846.33, 60 057.4}	12 081.2	1.86788	newly	437.	30 336.1	{3367., 73 663.}	6844.42	1.59411	Reff	1.563	0.617599	{0.25523, 1.36522}	60.8078	ar	63 119.	161 627.	{78 965., 256 118.}	156.514
20	R0	{1.54435, 5.32911}	{2.00407, 3.93467}	{{0.0896327, 0.93437}, {3., 5.82881}}	{78.7349, 42.0373}	inf	191.	4405.41	{512.009, 7240.31}	2206.95	1.102	newly	372.	5266.39	{449., 9570.}	1319.47	0.847938	Reff	1.53601	0.308997	{0.0133206, 0.450822}	79.8832	ar	61 870.	166 415.	{155 882., 182 900.}	171.222
21	R0	{1.57932, 4.90031}	{2.32547, 5.60863}	{{0.848292, 2.17253}, {3., 10.0947}}	{67.7662, 44.3582}	inf	57.	30 511.3	{11 900.1, 55 410.9}	53 428.9	2.56664	newly	102.	39 446.6	{12 292., 86 587.}	38 574.2	2.39955	Reff	1.57657	1.14655	{0.403839, 1.72333}	35.9443	ar	63 595.	137 637.	{79 301., 204 705.}	119.562
22	R0	{1.47044, 4.07694}	{2.3869, 5.26483}	{{1.76379, 1.27941}, {3., 12.8283}}	{62.3253, 85.1235}	inf	83.	30 152.	{4877.48, 60 617.3}	36 227.7	2.37331	newly	207.	31 918.7	{4918., 78 740.}	15 319.7	1.98911	Reff	1.46579	0.636905	{0.282678, 1.36596}	58.1571	ar	55 895.	166 776.	{100 987., 258 305.}	198.373
23	R0	{1.64997, 3.34546}	{2.16306, 9.06634}	{{0.578952, 2.10868}, {3., 12.8782}}	{58.1392, 184.206}	inf	9.	17 311.7	{3111.79, 36 754.}	192 253.	3.12111	newly	51.	23 218.2	{4304., 81 693.}	45 426.	2.38104	Reff	1.64943	1.57137	{0.274652, 2.80599}	38.2036	ar	66 404.	118 892.	{50 647., 189 919.}	89.8024
24	R0	{1.67405, 4.50254}	{1.7574, 8.01351}	{{0.372851, 2.00079}, {3., 16.7626}}	{55.2952, 126.188}	inf	14.	6899.46	{1102.83, 13 522.}	49 181.9	2.52399	newly	40.	21 909.9	{1593., 72 595.}	54 674.8	2.33297	Reff	1.6737	1.58365	{0.269689, 2.92653}	52.6907	ar	68 017.	68 746.2	{15 760., 106 672.}	49.8669
25	R0	{1.50663, 3.1286}	{1.72049, 8.04071}	{{0.315942, 2.00079}, {3., 16.7632}}	{63.4237, 187.054}	inf	3.	7332.14	{1102.2, 13 521.4}	244 305.	3.20833	newly	20.	20 402.4	{2042., 59 056.}	101 912.	2.63612	Reff	1.50652	1.54459	{0.297294, 2.9197}	58.2877	ar	58 823.	67 147.7	{15 910., 105 767.}	60.2525
26	R0	{1.69822, 6.34992}	{2.21533, 4.24483}	{{1.15883, 2.64583}, {3., 5.01104}}	{53.5732, 37.0371}	inf	141.	15 029.9	{8739.38, 37 874.4}	10 559.5	1.87734	newly	238.	16 541.6	{6672., 43 161.}	6850.24	1.66188	Reff	1.69328	0.37134	{0.0440735, 0.602215}	79.1777	ar	69 816.	157 287.	{65 113., 194 101.}	130.13
27	R0	{1.55869, 4.92988}	{2.41034, 4.76053}	{{1.77051, 1.25128}, {3., 12.8273}}	{58.3648, 69.6044}	inf	86.	25 689.9	{4021.47, 58 834.}	29 772.	2.26891	newly	214.	31 056.2	{2389., 78 615.}	14 412.2	1.89458	Reff	1.55537	0.576931	{0.101271, 1.36581}	63.6194	ar	62 344.	150 786.	{64 016., 257 377.}	146.215
28	R0	{1.46085, 6.06899}	{2.24008, 4.23442}	{{1.15729, 2.64636}, {3., 4.99632}}	{69.2689, 31.3355}	inf	547.	14 540.7	{8720.97, 41 489.7}	2558.59	1.28204	newly	795.	15 832.4	{6621., 56 158.}	1891.5	1.12212	Reff	1.43891	0.355	{0.0411993, 0.604758}	76.6986	ar	55 484.	167 121.	{123 542., 240 392.}	203.937
29	R0	{1.63017, 6.25164}	{2.50007, 4.73679}	{{1.77137, 1.22436}, {3., 12.8272}}	{55.8494, 70.8583}	inf	248.	25 184.4	{4843.7, 57 875.3}	10 056.8	1.80893	newly	360.	29 696.9	{4232., 73 354.}	8150.64	1.69437	Reff	1.6206	0.630604	{0.245828, 1.2814}	61.3276	ar	66 231.	156 854.	{92 372., 248 511.}	139.086
30	R0	{1.45173, 5.68165}	{2.40938, 6.84822}	{{1.46438, 3.38269}, {3., 10.2981}}	{65.9663, 49.1976}	inf	352.	3579.37	{1494.15, 8759.86}	920.503	0.760591	newly	541.	4903.31	{744., 25 418.}	809.991	0.545813	Reff	1.43681	0.609712	{0.0222071, 1.46324}	61.0042	ar	55 280.	185 904.	{170 341., 244 867.}	236.295
31	R0	{1.55808, 2.77316}	{1.72045, 7.89303}	{{0.315933, 2.00079}, {3., 16.4455}}	{60.2903, 208.686}	inf	4.	6944.52	{1101.99, 13 312.5}	173 513.	3.0718	newly	23.	20 935.8	{2022., 58 690.}	90 925.1	2.58633	Reff	1.55797	1.56527	{0.297294, 2.91973}	55.9024	ar	60 940.	67 512.3	{15 912., 105 343.}	57.8149
32	R0	{1.56797, 3.90327}	{2.26338, 10.4071}	{{1.15159, 1.99279}, {3., 17.0577}}	{57.3991, 177.066}	inf	24.	24 451.7	{3421.21, 45 193.6}	101 782.	2.8239	newly	62.	25 654.2	{3272., 61 135.}	41 277.8	2.3232	Reff	1.56717	1.41274	{0.383276, 2.22214}	32.5772	ar	62 228.	129 469.	{77 708., 177 659.}	110.607
33	R0	{1.70027, 5.43261}	{2.29747, 10.8986}	{{0.864734, 1.99295}, {3., 17.0636}}	{54.0335, 115.68}	inf	66.	26 650.6	{7819.76, 56 215.8}	40 279.8	2.48852	newly	163.	25 033.3	{6298., 64 972.}	15 257.9	1.9842	Reff	1.69787	1.37118	{0.264613, 2.22217}	36.3726	ar	68 798.	131 044.	{83 876., 171 807.}	91.5368
34	R0	{1.61668, 6.86192}	{2.09315, 8.42644}	{{0.420103, 2.71511}, {3., 13.7822}}	{69.384, 65.0726}	inf	325.	3296.23	{2798.18, 2798.18}	915.631	0.948235	newly	465.	2543.32	{1136., 4027.}	448.757	0.65776	Reff	1.60218	0.356988	{0.0315063, 0.650282}	78.0054	ar	64 693.	175 673.	{170 155., 181 385.}	171.549
35	R0	{1.55955, 3.45071}	{2.22059, 9.20413}	{{0.584094, 2.10869}, {3., 15.246}}	{62.9954, 184.507}	inf	11.	18 600.6	{5578.25, 36 750.9}	168 997.	3.09885	newly	53.	25 903.1	{4077., 81 772.}	48 773.8	2.4432	Reff	1.55925	1.53872	{0.27486, 2.1225}	40.5748	ar	61 783.	117 507.	{55 429., 182 647.}	95.5706
36	R0	{1.65068, 4.91307}	{2.18631, 6.36999}	{{0.847922, 2.17255}, {3., 10.0958}}	{62.1044, 56.684}	inf	26.	29 538.3	{8627.86, 50 909.6}	113 509.	2.92911	newly	80.	34 615.5	{9302., 63 789.}	43 169.4	2.47862	Reff	1.64972	1.06874	{0.394336, 2.08588}	44.4432	ar	66 453.	133 030.	{73 078., 198 476.}	104.967
37	R0	{1.46131, 3.06714}	{2.35605, 9.10698}	{{0.783557, 2.10859}, {3., 14.8339}}	{75.9242, 211.435}	inf	18.	18 547.8	{6810.78, 40 612.}	102 944.	2.89619	newly	72.	29 582.3	{4719., 84 246.}	40 986.5	2.38456	Reff	1.45985	1.82367	{0.380134, 2.99997}	51.5248	ar	56 452.	129 421.	{67 865., 182 712.}	131.972
38	R0	{1.71034, 5.2546}	{2.25736, 5.84627}	{{0.848001, 2.17249}, {3., 10.0953}}	{58.9238, 46.3344}	inf	60.	28 391.2	{8669.73, 53 966.8}	47 218.7	2.53446	newly	144.	35 357.3	{9584., 81 304.}	24 455.2	2.22914	Reff	1.70807	1.06068	{0.257534, 1.82027}	45.7037	ar	69 816.	126 723.	{70 398., 195 411.}	86.3089
39	R0	{1.43565, 5.57989}	{2.12081, 4.50874}	{{1.06051, 2.64641}, {3., 4.99793}}	{65.5542, 27.4642}	inf	400.	13 758.1	{4935.64, 34 511.}	3339.52	1.38594	newly	680.	14 862.5	{5042., 56 182.}	2085.66	1.14906	Reff	1.4178	0.342884	{0.0434671, 0.628428}	75.8159	ar	54 407.	163 410.	{78 880., 233 955.}	202.467
40	R0	{1.40327, 4.00764}	{2.07869, 6.21437}	{{0.849857, 2.17223}, {3., 10.0917}}	{73.3782, 78.8778}	inf	122.	30 731.3	{15 111.1, 52 662.2}	25 089.6	2.31002	newly	269.	36 037.4	{13 668., 69 823.}	13 296.8	2.02202	Reff	1.39731	1.05197	{0.404048, 1.5549}	35.0234	ar	51 976.	140 637.	{89 535., 199 314.}	170.581
41	R0	{1.63612, 4.26741}	{2.34058, 8.96355}	{{0.783627, 2.10869}, {3., 14.8324}}	{59.0546, 134.981}	inf	38.	17 357.9	{6664.6, 36 148.2}	45 578.8	2.54229	newly	108.	29 118.9	{4735., 83 879.}	26 861.9	2.19706	Reff	1.6346	1.85138	{0.460045, 2.99997}	39.0471	ar	65 029.	131 347.	{67 300., 182 776.}	106.688
42	R0	{1.57426, 6.50271}	{2.46562, 5.05265}	{{1.20964, 1.28827}, {3., 7.54648}}	{61.8428, 38.2921}	inf	287.	2767.88	{658.862, 2257.16}	866.064	0.667998	newly	463.	3550.2	{662., 4227.}	671.382	0.488594	Reff	1.56141	0.493849	{0.174745, 1.15203}	71.8223	ar	62 522.	181 010.	{167 443., 196 109.}	189.513
43	R0	{1.37818, 5.37636}	{2.42917, 6.48265}	{{1.46236, 3.3727}, {3., 10.2835}}	{76.2593, 52.3606}	inf	406.	4361.65	{1490.35, 8792.47}	977.563	0.718467	newly	728.	5165.95	{755., 25 347.}	613.328	0.450046	Reff	1.35702	0.584046	{0.0312684, 1.46182}	62.2856	ar	50 098.	187 069.	{170 009., 244 742.}	273.407
44	R0	{1.66606, 4.92641}	{2.26948, 10.3957}	{{0.853609, 1.99289}, {3., 17.058}}	{55.1124, 124.169}	inf	32.	25 525.4	{5315.21, 46 814.3}	79 666.9	2.74341	newly	79.	26 247.1	{4066., 61 517.}	33 124.1	2.25796	Reff	1.66501	1.36361	{0.288569, 2.22216}	36.0364	ar	67 066.	128 874.	{82 775., 171 448.}	94.0945
45	R0	{1.623, 5.9449}	{2.50281, 7.80533}	{{2.15899, 4.30553}, {3., 14.7091}}	{57.6871, 52.5784}	inf	133.	23 294.7	{6682.63, 43 463.2}	17 414.8	2.10649	newly	254.	23 101.4	{5699., 62 246.}	8995.05	1.77005	Reff	1.61652	0.869453	{0.328199, 2.1256}	54.6116	ar	64 554.	145 074.	{80 959., 220 849.}	126.068
46	R0	{1.65235, 5.51234}	{2.37168, 4.81443}	{{1.7602, 1.2492}, {3., 12.8241}}	{47.2753, 65.9748}	inf	70.	25 739.9	{4852.47, 58 175.3}	36 671.2	2.38297	newly	154.	30 916.1	{4895., 73 678.}	19 975.4	2.08333	Reff	1.64944	0.617208	{0.161405, 1.36588}	63.6137	ar	66 509.	155 275.	{68 801., 258 290.}	138.163
47	R0	{1.62138, 8.82208}	{2.52654, 7.08511}	{{1.4576, 3.36768}, {3., 10.2541}}	{59.454, 35.1738}	inf	1152.	3148.72	{1485.83, 2797.21}	176.869	0.203828	newly	1173.	3131.19	{736., 3324.}	195.859	0.255876	Reff	1.57308	0.506417	{0.0276869, 0.712824}	70.1864	ar	65 598.	179 801.	{169 959., 187 670.}	174.096
48	R0	{1.64073, 6.69261}	{2.48496, 7.64575}	{{2.16097, 4.30492}, {3., 14.7095}}	{52.7854, 47.5607}	inf	217.	22 539.4	{5333.36, 42 926.3}	10 286.8	1.87673	newly	314.	25 955.2	{6219., 71 641.}	8165.98	1.71806	Reff	1.63171	1.10048	{0.442924, 2.18581}	49.7014	ar	66 438.	160 416.	{99 582., 229 473.}	141.86
49	R0	{1.65556, 3.796}	{1.73238, 7.85575}	{{0.31596, 2.00079}, {3., 16.7628}}	{55.7343, 150.016}	inf	5.	6831.7	{1102.62, 13 313.1}	136 534.	2.97352	newly	9.	22 167.2	{1987., 72 659.}	246 202.	3.00837	Reff	1.65538	1.57201	{0.269684, 2.92656}	53.6708	ar	66 575.	67 965.1	{15 849., 106 373.}	50.6435
50	R0	{1.56828, 3.65713}	{2.22205, 8.97799}	{{0.552281, 2.10868}, {3., 13.6352}}	{61.5529, 162.755}	inf	37.	18 132.5	{6517.03, 36 648.7}	48 906.7	2.58228	newly	103.	25 491.5	{4722., 81 861.}	24 649.	2.18651	Reff	1.56703	1.68243	{0.39418, 2.99997}	42.5733	ar	62 761.	124 917.	{55 682., 189 113.}	104.304
